# Left main and two vessels calcified coronary aneurysms presented as out of hospital cardiac arrest in young patient

**DOI:** 10.1002/ccr3.6398

**Published:** 2022-10-03

**Authors:** Khaled Al Khodari, Raad Alhaj Tahtouh, Abdulrahman Arabi, Mouaz Al Khodari

**Affiliations:** ^1^ Cardiology Department Hamad Medical Corporation (HMC) Doha Qatar; ^2^ Internal Medicine Department Hamad Medical Corporation (HMC) Doha Qatar; ^3^ School of Medicine Syrian Private University Damascus Syria

**Keywords:** coronary angiogram, coronary artery aneurysms, coronary artery bypass graft surgery, out of hospital cardiac arrest

## Abstract

A 37‐year‐old patient was admitted secondary to ventricular fibrillation induced out of hospital cardiac arrest. Coronary angiogram revealed left main, left anterior descending, and right coronary arteries aneurysms. The patient underwent bypass surgery with four grafts. Ejection fraction improved from 30% upon admission to 45% at 3 months of follow‐up.

## Abstract 2

Coronary artery aneurysms are usually asymptomatic; however, cardiac arrest or sudden cardiac death might be the initial presentation for such vascular anomalies. The management is challenging and individualized because of the absence of evidence‐based guidelines, which necessitates the need for adequately powered randomized clinical trials to guide treatment strategy.

## 1 INTRODUCTION

Coronary artery aneurysm (CAA) is an uncommon vascular anomaly that is defined as local dilatation of coronary artery that exceeds 1.5 times the adjacent normal segment.^1^ It differs from coronary artery ectasia (CAE) as the last is associated with diffuse dilation of coronary artery involving more than a third of coronary artery length.^2^, ^3^ The incidence ranges from 1.5% to 5%, and it has male predominance with preference of right coronary artery (RCA).^1^, ^4^ Left main (LM) aneurysm is extremely rare and is found in 0.1% of patients undergoing coronary angiography.^5^

Atherosclerosis is the most common cause of CAA in adult patients. It accounts for 50% of all cases, whereas CAA complicates Kawasaki disease in children. Other causes of CAA in adults are congenital (second most common), inflammatory and connective tissue disorders, infections, cocaine intoxications, traumatic, and iatrogenic, such as percutaneous coronary intervention (PCI).^4^, ^6^

Most coronary aneurysms are asymptomatic and detected incidentally. However, they might present with acute myocardial infarction, exertional angina, local thrombosis and distal embolization, massive enlargement and compression of adjacent structures, fistula formation, or expansion in size followed by aneurysmal rupture with subsequent pericardial tamponade or sudden death.^7^, ^8^

Our patient had an extensive aneurysmal coronary disease resulting in myocardial ischemia with subsequent drop of ejection fraction (EF) and myocardial scarring. He presented with out of hospital cardiac arrest (OHCA) secondary to ventricular fibrillation (VF) that responded to direct current (DC) shocks.

## 2 CASE PRESENTATION

**FIGURE 1:**
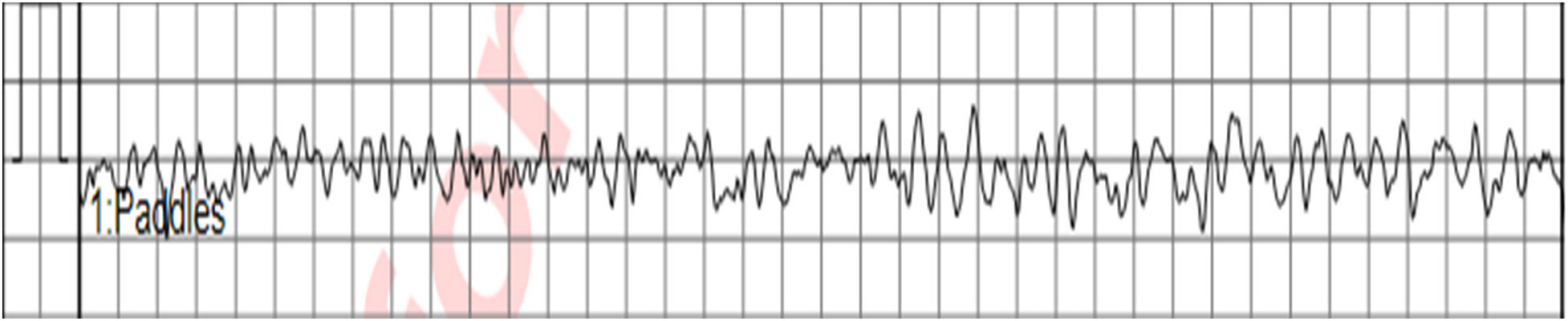
Initial Rhythm strip upon emergency medical services (EMS) team first encountered the patient, it showed ventricular fibrillation (VF).

A previously healthy 37‐year‐old male patient admitted to cardiology intensive care unit (CICU) as a case of out of hospital cardiac arrest (OHCA). The history began, as per his office colleagues who witnessed the event, when he complained of headache and dizziness then he collapsed suddenly after few minutes and became unresponsive. Bystander cardiopulmonary resuscitation (CPR) was started immediately, and emergency medical services (EMS) team came within 5 min. Initial rhythm was ventricular fibrillation (VF), (Figure 1), that was shocked twice and return of spontaneous circulation (ROSC) was achieved. Total CPR time was around 10 min. After that, the patient was intubated because of poor respiratory effort and shifted to emergency department (ED).

**FIGURE 2:**
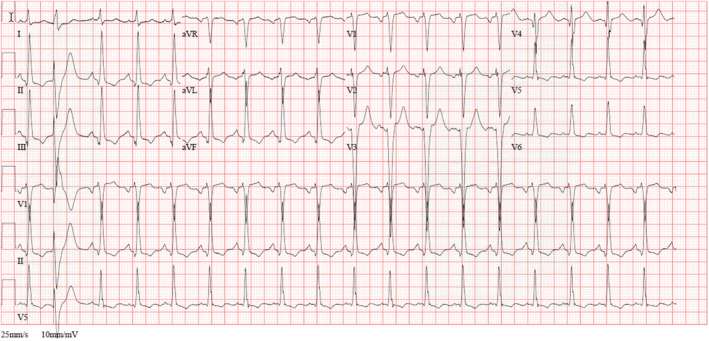
Electrocardiogram (ECG) after return of spontaneous circulation (ROSC) revealed sinus rhythm with signs of left ventricular hypertrophy. Second beat was a premature ventricular beat (PVC). There were no ST segment changes suggestive of acute coronary syndrome (ACS).

In ED, the patient was sedated with midazolam infusion and intubated on continuous mandatory ventilation (CMV) mode. Noradrenaline infusion was started to support blood pressure. Electrocardiogram (ECG) revealed left ventricular hypertrophy (LVH) (Figure 2). Initial lactic acid was 4.6 mmol/L, PH was 7.16, PaO2 and PaCO2 were 105 mmHg and 85 mmHg, respectively. Complete blood count (CBC), electrolytes, renal and liver function testes were within normal range. High sensitivity troponin T was 67 ng/L (normal <14 ng/L). Computed tomography (CT) scan ruled out acute central nervous system insult and pulmonary embolism. Echocardiography exhibited depressed EF (30%) with apical akinesia and all other segments were hypokinetic. Acute coronary syndrome (ACS) was unlikely because the patient was not complaining of chest pain before collapse and no clear ischemic changes on ECG with mildly raised troponin. Next day, the patient extubated successfully, noradrenaline tapered down. However, the patient had another cardiac arrest secondary to pulseless ventricular tachycardia (VT). Two DC shocks were given, and ROSC was achieved after 4 min then he was awake and stable, and amiodarone infusion was initiated.

**FIGURE 3:**
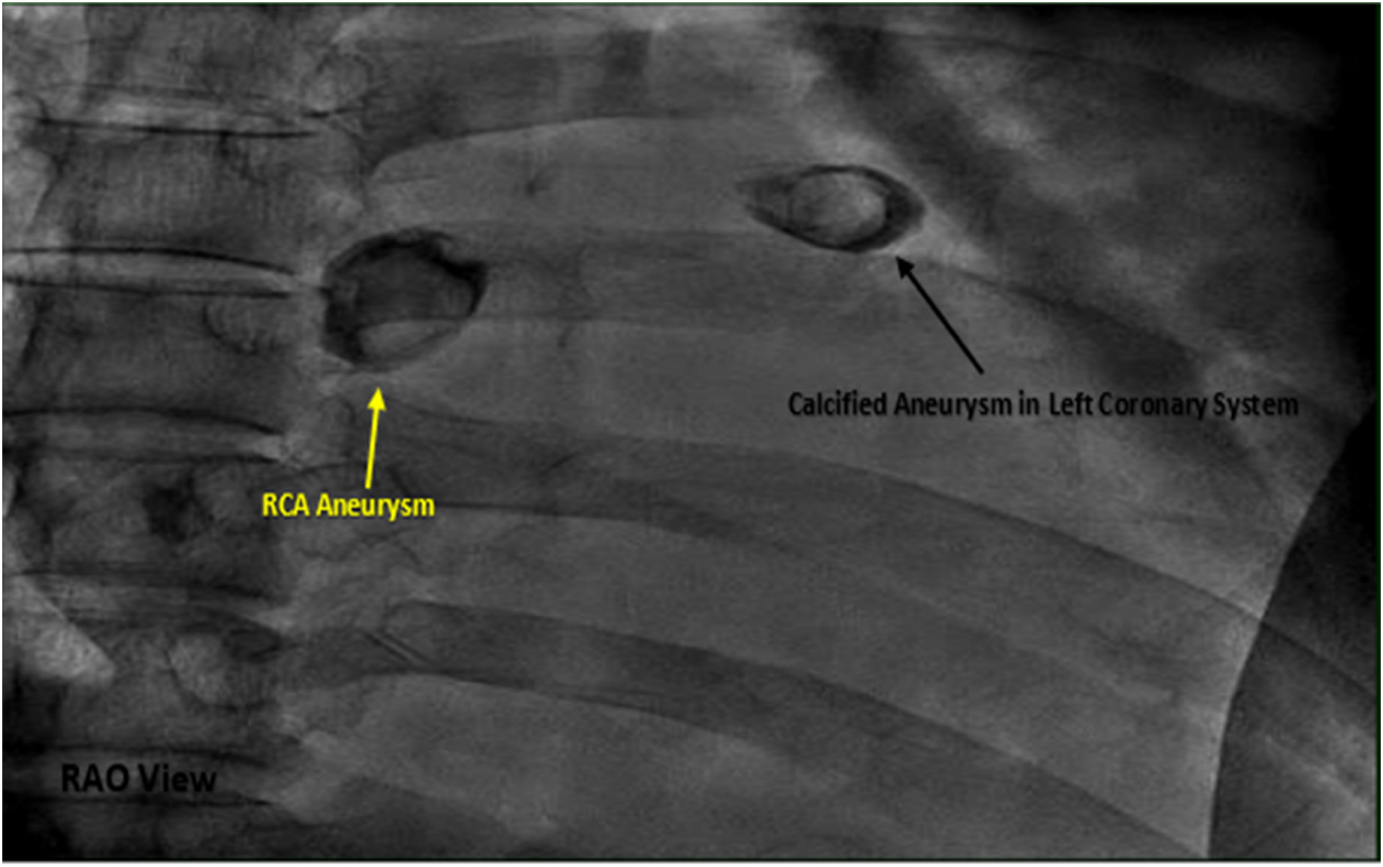
Fluoroscopic image in right anterior oblique (RAO) projection before coronary engagement showing heavily calcified giant aneurysms in left (black arrow) and right (yellow arrow) coronary systems.

**FIGURE 4:**
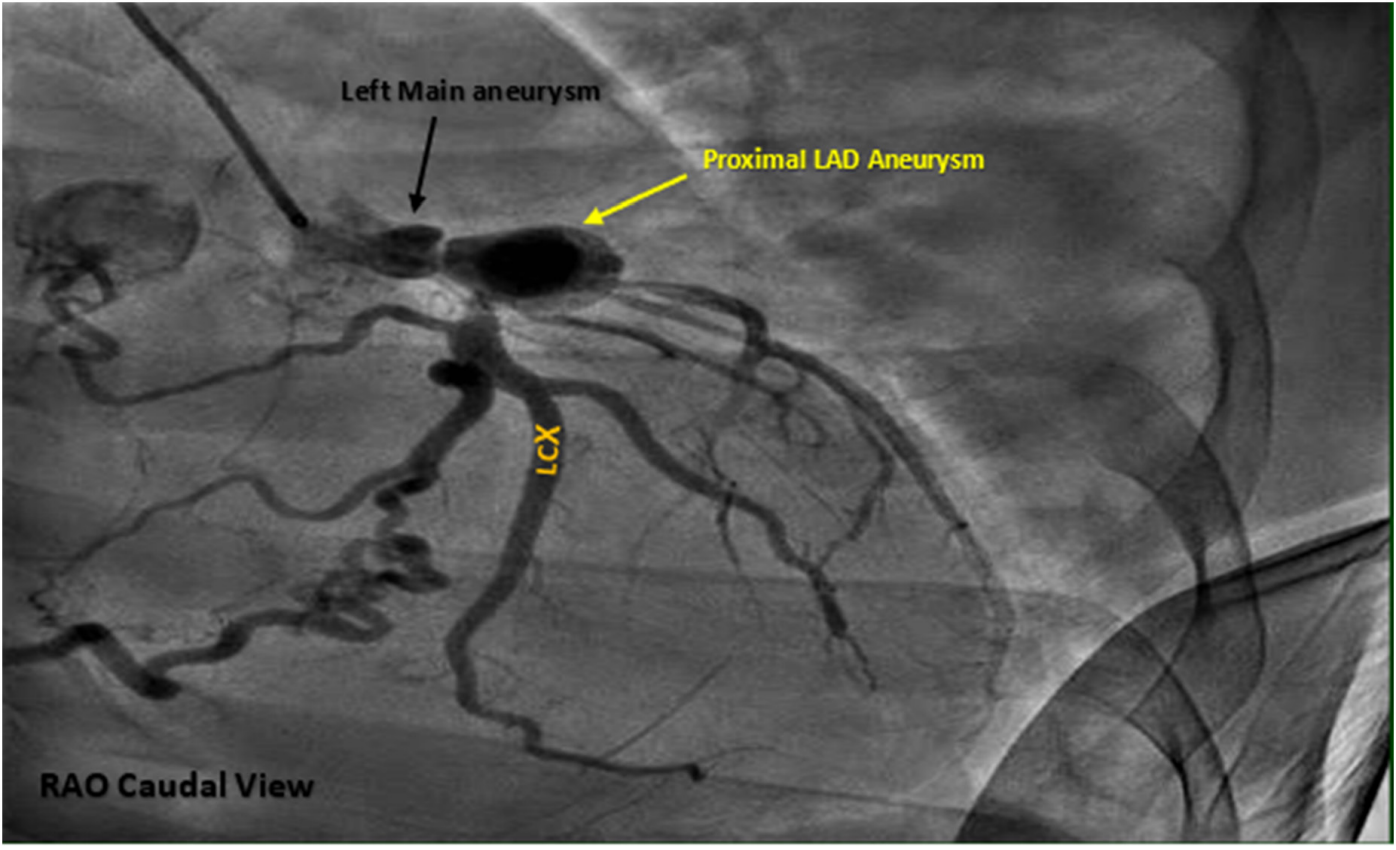
Fluoroscopic image in right anterior oblique (RAO) caudal projection of left coronary system. It revealed left main (LM) aneurysm (black arrow) followed by critical distal LM stenosis 90% and proximal left anterior descending (LAD) artery aneurysm (yellow arrow) with subsequent proximal LAD 100% stenosis. It revealed also tight stenosis of proximal left circumflex (LCX).

**FIGURE 5:**
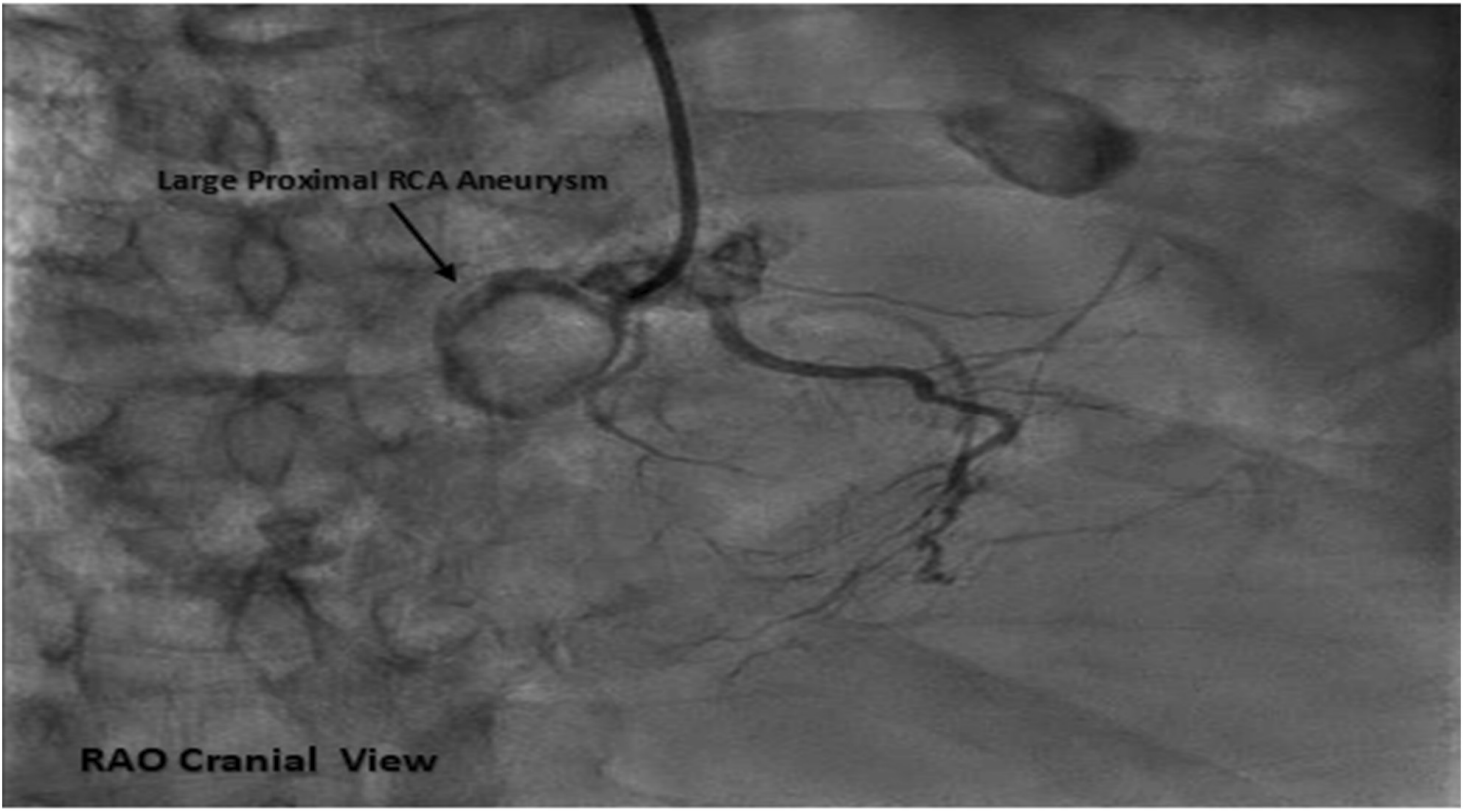
Fluoroscopic image in right anterior oblique (RAO) cranial projection of right coronary artery (RCA) exhibited large aneurysm (arrow) in proximal RCA with subsequent 100% occlusion.

Coronary angiogram (CAG) revealed coronary aneurysms in left main (LM), proximal left anterior descending (LAD) artery, and right coronary artery (RCA) with subsequent critical stenosis in LM and proximal segments in LAD, RCA, and left circumflex artery (LCX) (Figures 3, 4, 5). It exhibited also bridging collaterals between left and right systems.

Urgent bypass surgery was performed next day with four bypass grafts. Left internal mammary artery (LIMA) to LAD, saphenous vein grafts (SVG) to obtuse marginal (OM), posterior descending artery (PDA), and ramus.

Extensive work up for vasculitis, autoimmune, and connective tissue diseases, including positron emission tomography (PET) scan and aortic wall biopsy, were negative. Postoperatively, the patient continued to have episodes of VT, without hemodynamic instability, which had poor response to amiodarone and beta blockers. Cardiac magnetic resonance imaging revealed picture of ischemic cardiomyopathy and apical scarring; therefore, an implantable cardioverter‐defibrillator (ICD) device was placed. The patient was discharged in stable condition.

The patient was totally asymptomatic during outpatient follow‐up, and he denied receiving any shocks from ICD device. EF improved to 45% with repeated echocardiography 3 months after discharge. No further runs of life‐threatening ventricular arrhythmia were detected by device interrogation in outpatient follow‐up.

## 3 DISCUSSION

Coronary artery aneurysms (CAAs) can be classified based on morphology, vessel wall structure, and size. They are considered saccular when transverse >longitudinal diameter, whereas fusiform ones are the opposite. True aneurysms involve all three vessel layers with preserved vessel wall integrity; However, pseudoaneurysm is characterized by loss of wall integrity and damaged adventitia. When the diameter is more than 20 mm, giant aneurysm is defined.^8^, ^9^

The pathophysiology of CAA is not well established. It is believed to be the same to that for large vessels aneurysm with injury and disfigurement of vessel media leading to increased wall stress and subsequent dilatation.^6^ The strong relationship between CAA and atherosclerotic cardiovascular disease indicates that both diseases might share the same risk factors and etiology.^8^, ^10^ Other possible mechanisms of CAA include genetic predisposition in idiopathic CAA, autoimmune/inflammatory process in vasculitis and connective tissue diseases, direct vessel wall injury in iatrogenic ACC, for example, post‐coronary stenting, dynamic increase in wall stress in patients with cocaine abuse, and infectious by direct invasion of vessel wall (mycotic aneurysm) or immune complex deposition.^8^, ^11^, ^12^

The evidence behind the best management of CAA is scarce because of the absence of randomized trials or large‐scale data. It is primarily based on case reports or small case series.^4^, ^8^ What makes it more challenging is that the uncertainty about the need to treat incidentally discovered CAA in asymptomatic patients without associated coronary stenosis. Furthermore, the technical difficulties that might we face when surgical or percutaneous intervention is warranted. Consequently, the treatment should be individualized based on clinical presentation, CAA location and morphology, and patient's clinical characteristics.^8^

Treatment options consist of medical therapy, percutaneous, and/or surgical intervention. Up to now, there has been no strong evidence to support the use of certain medications in adult patients with CAA.^8^, ^11^ If there is a high suspicion of thrombus and/or embolism, long‐term anti‐coagulation might be considered.^10^ Intravenous immunoglobulin (IVIG) should be considered in patients with CAA secondary to Kawasaki disease. Early initiation of IVIG and smaller CAA at time of diagnosis, have been related to lower rate of major adverse‐coronary events and higher chance for CAA complete regression.^13^

Percutaneous intervention using covered stents is a new treatment modality that can be used in certain patients with CAA. It has some limitation, including the risk of restenosis, stent thrombosis, occlusion of side branch, or no reflow after implementation. Thus, their use is restricted to specific cases only with small CAA (<10 mm).^14^ Surgical intervention should be considered in patients with associated obstructive coronary artery disease, large saccular aneurysms (>10 mm), high risk of rupture, or in whom percutaneous intervention is not appropriate. Surgical options include bypass surgery, the most common, aneurysmal resection, ligation, or marsupialization with interposition graft.^15^ Ideal surgical intervention has not been yet identified.

Our patient was treated with bypass surgery because he had an extensive CAA involving left main, proximal LAD, and RCA with subsequent severe stenosis. Upon outpatient follow‐up, he was asymptomatic without any limitation in daily activities.

## 4 CONCLUSION

Coronary artery aneurysms are not completely benign entities because they might have fatal presentations and/or outcomes similar to our patient. The management is challenging and individualized based on clinical presentation, aneurysm size, and location. Our case necessitates the need for conducting large randomized clinical trials to obtain evidence‐based guidelines which can help in dealing with such patients.

## AUTHOR CONTRIBUTIONS

All authors were involved in the conception and design, critical revision, manuscript writing, final approval, and agreed to be accountable for all aspects of the work.

## CONFLICT OF INTEREST

The authors have no relationships relevant to the contents of the paper to disclose.

## ETHICAL APPROVAL

Ethical approval to report this case was obtained from Hamad Medical Corporation (HMC) Ethics Committee; ID: MRC‐04‐22‐378.

## CONSENT

Written informed consent was obtained from the patient to publish this report in accordance with the journal's patient consent policy.

## Data Availability

The data that support the findings of this study are available from the corresponding author, Khaled Al khodari, upon reasonable request.
